# Identification of immune-related diagnostic genes and immune cell infiltration in aseptic loosening of prostheses after total hip arthroplasty by integrated bioinformatics analysis and experimental confirmation

**DOI:** 10.3389/fgene.2025.1597219

**Published:** 2025-08-05

**Authors:** Yunke Liu, Xiaming Liang, Shuo Qiang, Yonghui Dong, Xin Zhao, Lichao Ma, Zhihua Yan, Songkai Yue, Yifan Huang, Jia Zheng

**Affiliations:** Department of Orthopedics, Henan Provincial People’s Hospital, People’s Hospital of Zhengzhou University, Zhengzhou, Henan, China

**Keywords:** aseptic loosening, total hip arthroplasty, macrophages, bioinformatics, immune infiltration

## Abstract

**Background:**

Aseptic loosening (AL) of hip prostheses is one of the main reasons for revision total hip arthroplasty (rTHA). However, the transcriptomic characteristics of AL are scarcely understood. This study aimed to discover candidate biomarkers for the diagnosis of AL.

**Patients and methods:**

The interface membrane from four patients with AL of hip prostheses and the synovium samples from four patients with a periprosthetic femoral fracture (PFF) after total hip arthroplasty (THA) were analyzed via RNA sequencing. Integrated bioinformatics analysis was employed to identify immune-related hub genes in AL. Immune cell infiltration analysis and correlation analysis were performed. Connectivity map analysis was utilized to predict the potential small-molecule compounds for AL treatment. Western blotting and histological staining were used to verify the expression of hub genes in AL.

**Results:**

A total of 2,184 differentially expressed genes (DEGs) were identified in the AL samples, including 2,050 upregulated genes and 134 downregulated genes, and these DEGs were mainly enriched in immune cell-related signaling pathways and immune-related processes. Immune cell infiltration analysis showed that the proportion of M1 macrophages increased in AL. Three genes closely related to M1 macrophages were screened, namely, *CD68*, *CD163*, and *SPP1*, according to the results of correlation analysis. Hematoxylin–eosin staining showed that the synovitis score of AL samples was significantly higher than that of controls (average, 6.2 vs. 3.8). Western blotting and immunohistochemical analysis showed that the expression of *CD68*, *CD163*, and *SPP1* in the AL group was significantly higher than that in the control group. The top 10 compounds with the highest negative scores were predicted to be potential therapeutic drugs for the treatment of AL.

**Conclusion:**

Preliminary transcriptomic signatures suggested that *CD68*, *CD163*, and *SPP1* may serve as potential biomarkers for AL, offering a novel research perspective for future diagnosis and therapeutic intervention of AL.

## Introduction

Revision total hip arthroplasty (rTHA) is a procedure in which the prosthesis fails for various reasons after the primary total hip arthroplasty (THA) and another operation is required to remove or replace the prosthesis. With the increase in the number of primary THA, the demand for rTHA continues to increase in nearly 20 years (Kurtz et al., 2007). However, the incidence of complications after revision surgery is higher, which was twice that of complications after primary THA, and the possibility of further re-revision was five times that of patients receiving primary THA (Mahomed et al., 2003; Badarudeen et al., 2017). Revision surgery places a huge physical and emotional burden on patients and increases financial pressure on families, society, and healthcare facilities (Murphy et al., 2024; Ashkenazi et al., 2023). The main reasons for rTHA are prosthesis instability, periprosthetic infection, and aseptic loosening (AL) of the prosthesis (Melvin et al., 2014). Currently, the diagnosis of instability is relatively clear; nevertheless, a large amount of research is focused on improving the diagnosis and treatment of periprosthetic infections. However, AL remains a diagnostic challenge (Anil et al., 2022). AL occurs when there is no sign of infection in the bone surrounding the prosthesis, but the bond between the prosthesis and the surrounding bone tissue is weakened, resulting in displacement or instability of the prosthesis (Abu-Amer et al., 2007). AL can be caused by a variety of factors, such as wear and tear of the prosthesis material, bone resorption, improper prosthesis design, surgical techniques, patient activity level, and osteoporosis (Cherian et al., 2015).

One of the most common theories regarding the pathogenesis of AL is that the formation of excessive wear particles creates a pro-inflammatory state, leading to increased osteoclast differentiation and macrophage production, which ultimately results in local bone resorption around the prosthesis and AL (Jiang et al., 2013). Macrophages play a crucial role in the pathogenesis of AL, including the regulation of inflammatory responses and associated pathological bone remodeling. The polarization of macrophages is closely linked to the microenvironment surrounding the prosthesis. Classically activated M1 macrophages are known for their increased production of pro-inflammatory cytokines, whereas activated M2 macrophages primarily function in resolving inflammation and promoting tissue repair (Cong et al., 2023). Consequently, investigating the molecular characteristics of macrophage-mediated AL of prostheses holds significant clinical importance for the prevention and treatment of AL in the future.

In recent years, integrated bioinformatics analysis has emerged as a powerful tool for identifying new genes associated with various diseases, which can act as biomarkers for disease diagnosis (Rhodes and Chinnaiyan, 2005). Previous studies have revealed the expression patterns of circular RNAs, the situation of ceRNA networks, and the expression patterns of exosomal microRNAs in the pathogenesis of AL (Ni et al., 2021; Pan et al., 2023). However, there is still limited research on identifying pathogenic genes that can act as potential biomarkers for AL of prostheses. Thus, the aim of this study is to identify key genes associated with AL of prostheses, offering new perspectives for the diagnosis, treatment, and investigation of potential pathogenic mechanisms of AL.

In this study, we obtained the interface membrane from the vicinity of prostheses in AL patients and used synovium samples from patients with a periprosthetic femoral fracture (PFF) after THA as a control group. We employed mRNA sequencing analysis to generate the gene expression profile of AL samples and leveraged various integrated bioinformatics tools, such as differentially expressed gene (DEG) analysis, functional enrichment analysis, immune infiltration analysis, and potential small-molecule compound prediction. Ultimately, preliminary transcriptomic signatures identified the key genes *CD68*, *CD163*, and *SPP1*, which are associated with macrophage-mediated immune regulatory processes, and validated their expression in AL samples.

## Materials and methods

### Acquisition of specimens

We selected patients with periprosthetic AL after primary THA who underwent rTHA from May 2023 to May 2024 at the Department of Orthopedics, Henan Provincial People’s Hospital, as the study group. The diagnosis of AL was consistent with the diagnostic criteria described in previous literature. Seven patients with a PFF after THA from our hospital were recruited as the control group, with the diagnosis of PFF conforming to the Unified Classification System (UCS) (Duncan and Haddad, 2014). Informed consent was obtained from all participants or their families. This study was conducted in accordance with the principles of the Declaration of Helsinki (1975) and was approved by the Medical Ethics Committee of Henan Provincial People’s Hospital (IRB ID: 2022-68). The interface membrane from the AL group was obtained from the surrounding tissues of the liner–ball–prosthetic neck, whereas the control samples were obtained from the hyperplastic synovial tissue within the hip joint adjacent to the prosthesis, matching the AL group’s anatomical origin. Demographic data such as gender, age, body mass index (BMI), smoking history, and drinking history were recorded for both groups, and in addition, data on the prosthetic survival period after primary THA, diagnosis, site of prosthetic loosening, and type of implant used for primary THA were also recorded for the AL group.

### RNA sequencing

Sample preparation: the interface membrane from the AL group and the synovial tissues from the control group were fully ground using a homogenizer. Total RNA is extracted from the samples using TRIzol (Invitrogen). The mRNA is then isolated from the total RNA using Oligo-dT magnetic beads (Invitrogen).

Fragmentation: the purified mRNA is fragmented into smaller pieces of ∼300 bp.

cDNA synthesis: the fragmented mRNA is reverse-transcribed into cDNA using cDNA synthesis reagents (Yeasen Biotechnology).

Adapter ligation: adapters are ligated to both ends of the cDNA fragments.

cDNA purification and fragment sorting: beads are utilized to selectively bind and isolate the 200–300 bp of DNA fragments (Yeasen Biotechnology).

Amplification: the adapter-ligated cDNA fragments are amplified using PCR to generate sufficient material for sequencing (Thermo Fisher Scientific).

Sequencing: the amplified cDNA library is loaded onto a flow cell and sequenced using the Illumina NovaSeq X Plus platform (Illumina, United States).

### Principal component analysis (PCA)

Sangerbox tools (http://www.sangerbox.com/tool) is a user-friendly interface that supports differential analysis and provides interactive customizable analysis tools, including PCA, various types of correlation analyses, and enrichment analyses, as well as some other common tools and functions (Shen et al., 2022). The gene expression profile data obtained from RNA sequencing were analyzed using the “stats” package in R software. Specifically, the z-score was first performed on the expression profile, and the “prcomp” function was further used for dimensionality reduction analysis to obtain the matrix after dimensionality reduction.

### Differentially expressed gene analysis

The gene expression profile data were analyzed using the “limma” package in R software to identify DEGs; |log2FC| ≥ 1 and adjusted *p*-value <0.05 were considered statistically significant. The expression patterns of DEGs were visualized in the form of volcano plots and heatmaps.

### Functional enrichment analyses

The DEGs were imported into Sangerbox tools, and Kyoto Encyclopedia of Genes and Genomes (KEGG) enrichment analyses and Gene Ontology (GO) enrichment analyses, including biological process (BP), molecular function (MF), and cellular component (CC), were completed based on the latest subset gene annotation. An adjusted *p-*value <0.05 and FDR < 0.05 were considered indicative of significant signaling pathways. The enrichment analysis results were visualized using bubble plots or Circos plots.

### Protein–protein interaction network analysis and cluster analysis

The top 250 DEGs were selected using the STRING database (https://string-db.org) for protein–protein interaction (PPI) network analysis, and the data were visualized using Cytoscape software and its Molecular Complex Detection (MCODE) plugin (version 3.8.2, San Diego, United States). The MCODE function in Cytoscape was further utilized to screen gene clusters, which are ranked according to their scores.

### Immune cell infiltration analysis

Gene expression profile data were analyzed using Sangerbox tools immunoinfiltration analysis function. The R software “CIBERSORT” package was used to calculate the proportion of 22 types of immune cells in each sample with the Wilcoxon test (Newman et al., 2015); a *p-*value <0.05 was considered statistically significant, and stacked histogram visualization was performed. Spearman’s correlation coefficient was employed to analyze the correlation between the proportion of infiltrating immune cells and immune-related DEGs; a *p-*value <0.05 or −log (*p-*value) >1.3 was considered statistically significant, and a heatmap was used for visualization.

### Western blotting

The interface membrane and synovial tissues of the same quality were cut into 1-mm^3^ pieces using eye scissors, fully cracked at 4°C for 30 min according to the ratio of 20 mg tissue:200 μL lysate, and completely shaken once every 10 min. The cracked sample was centrifuged at 14,000g at 4°C for 10 min, the supernatant retained, the protein concentration determined, and the protein concentration of different samples adjusted to be consistent. Western blot analysis was performed after the loading buffer was added. The following primary antibodies were used: anti-CD68 (1:4,000, Proteintech), anti-CD163 (1:500, Proteintech), anti-OPN (1:2,000, Proteintech), and anti-β-actin (1:1,000, PTM). The intensity of each band was quantified using AlphaEaseFC software, and the expression was calculated relative to the β-actin level.

### Histological staining

The interface membrane and synovial samples were fixed with 10% neutral formalin, and paraffin sections were performed. After dehydration, hematoxylin–eosin (HE) staining was carried out to evaluate the inflammatory infiltration of synovial tissue by the synovitis score (Krenn et al., 2006). For immunohistochemical (IHC) analysis, after dehydration, the paraffin sections were repaired with citric acid, and endogenous peroxidase blocker was added to seal the sections with bovine serum albumin. The following primary antibodies were used: anti-CD68 (1:2,000, Proteintech), anti-CD163 (1:1,000, Proteintech), and anti-OPN (1:250, Proteintech). The average optical density (AOD) of CD68, CD163, and OPN was statistically analyzed using ImageJ (version 1.8.0). AOD (%area) = integrated option density (IOD)/area.

### Connectivity map analysis

The top 150 upregulated DEGs were screened using the criteria of |log_2_FC| > 1.5 and adjusted *p-*value <0.05 and then were analyzed using the CMap database (https://clue.io) to search for potential small-molecule compounds. The top 10 small-molecule compounds with the highest negative connectivity scores (CSs) were identified as small-molecule compounds with potential therapeutic effects, and the results were visualized in the form of heatmaps and Sankey maps.

### Statistical analysis

All data between the two groups were analyzed statistically and plotted using GraphPad Prism software (version 8.0, San Diego). The categorical variables were assessed using Fisher’s test. Descriptive statistics are expressed as mean with standard deviation. The normality of the data distribution was evaluated using the Kolmogorov–Smirnov test. Levene’s test was used to assess the homogeneity of variance. Independent samples *t*-tests were applied to analyze normally distributed values. Data with non-Gaussian distribution were analyzed using the non-parametric Mann–Whitney *U* test. Two-tailed values of *p* < 0.05 were considered statistically significant.

## Results

### Demographic characteristics of AL patients and controls

A total of five patients with AL of hip prostheses and five patients with a PFF after THA were studied. Three patients in the AL group had the diagnosis of avascular necrosis of the femoral head (ANFH) at primary THA, and two patients had a fracture of the femoral neck. The control group is the same as the AL group. The frictional interface of the primary THA prostheses in both AL and control groups includes two cases of ceramic–polyethylene and three cases of ceramic–ceramic bearings. The mean survival time of the prosthesis in the AL group was 6.8 ± 2.59 years when rTHA was performed, including one case of simple acetabular prosthesis loosening, two cases of simple femoral stalk prosthesis loosening, and two cases of both acetabular and femoral prosthesis loosening. The mean survival time of the prosthesis in the control group was 4.6 ± 3.13 years. There were no significant differences in age, sex, BMI, operative site, friction interface, and drinking and smoking history between the two groups. The demographics of the two groups are shown in Table 1.

**TABLE 1:** Demographics of the AL patients and controls.

Group	Control	AL	*p*-value
Number	5	5	-
Gender (male/female)	3/2	2/3	>0.9999
Age (year)	65.40 ± 5.18	67.80 ± 4.60	0.6032
BMI	25.28 ± 1.42	25.58 ± 1.61	0.6905
Operative site (left hip/right hip)	3/2	4/1	>0.9999
Preoperative diagnosis	Periprosthetic fracture after THA	Aseptic loosening of prostheses after THA	-
Preoperative diagnosis of primary THA	Avascular necrosis of the femoral head/fracture of the neck of the femur (3/2)	Avascular necrosis of the femoral head/fracture of the neck of the femur (3/2)	>0.9999
Types of surgery	Primary THA	Revision THA	-
Prosthesis survival after primary THA (year)	4.6 ± 3.13	6.8 ± 2.59	0.2937
Prosthetic loosening site	Acetabular side/femur side (0/5)	Acetabular side/femur side (3/4)	0.2045
Friction interface of primary THA	Ceramic–polyethylene/ceramic–ceramic (2/3)	Ceramic–polyethylene/ceramic–ceramic (2/3)	>0.9999
Drinking history (yes/no)	2/3	2/3	>0.9999
Smoking history (yes/no)	1/4	2/3	>0.9999

Data are numbers or the mean ± SD.

BMI, body mass index.

### Identification of DEGs for the diagnosis of AL

**FIGURE 1:**
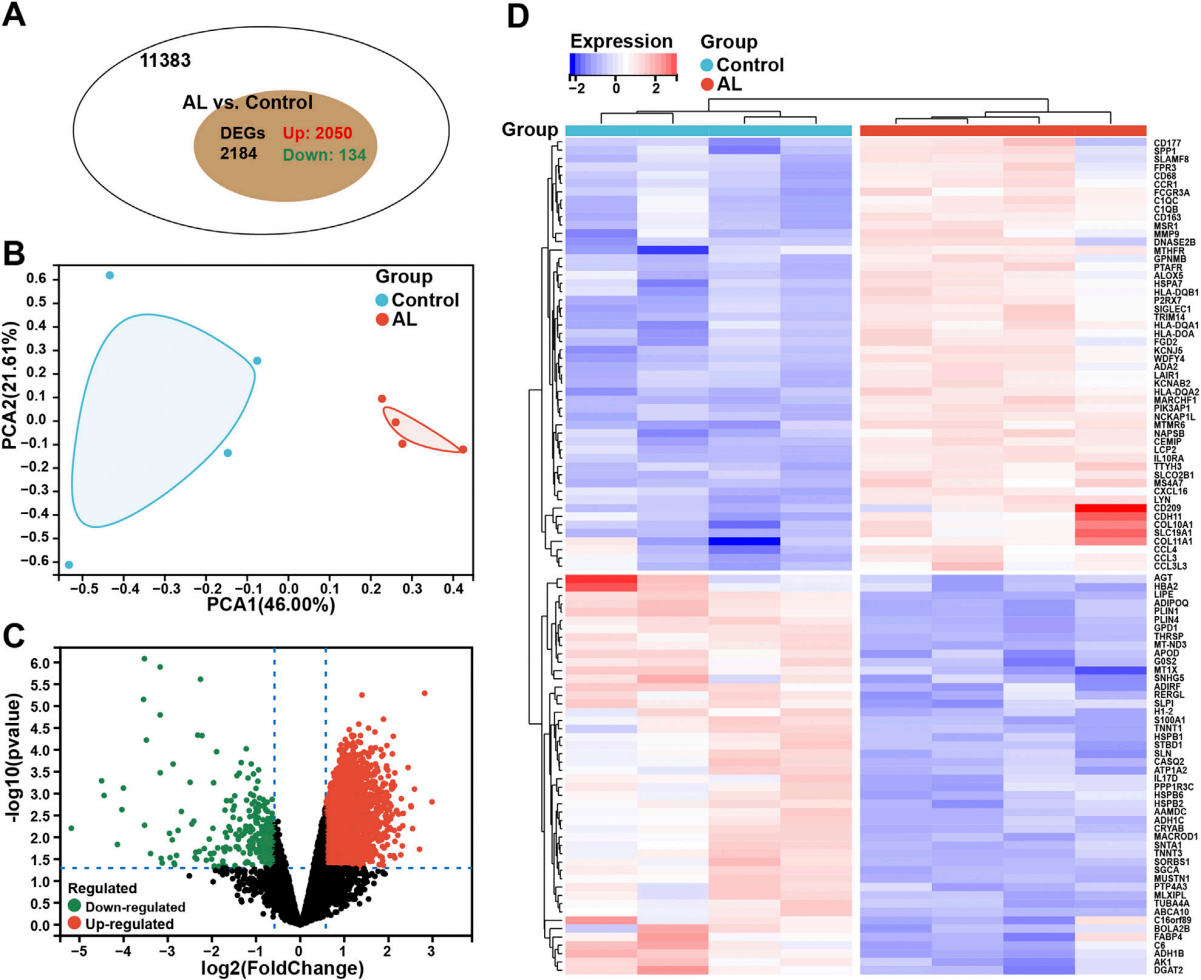
Identification of DEGs for the diagnosis of AL. **(A)** Venn chart representing the total number of genes and the number of DEGs in the AL samples (n = 4), with red representing upregulated genes and green representing downregulated genes. **(B)** PCA was performed to classify the samples as the AL and control groups. **(C)** Volcano plots representing the expression pattern of AL DEGs, with red representing upregulated genes, green representing downregulated genes, and black representing genes with no significant differences. **(D)** Heatmap representing the expression patterns of the top 50 significantly upregulated or downregulated DEGs in the AL samples, with each row representing a DEG and each column representing a sample of AL cases or controls.

A total of 2,184 DEGs were identified in AL samples with an adjusted *p*-value <0.05, including 2,050 upregulated genes and 134 downregulated genes (Figure 1A). The PCA results showed significant differences between the two samples within the group (Figure 1B). The volcano plot was used to describe the expression pattern of DEGs in the AL samples and controls (Figure 1C), and the heatmap showed the top 50 upregulated DEGs and the top 50 downregulated DEGs (Figure 1D).

### Enrichment analysis and PPI network analysis of AL samples

**FIGURE 2:**
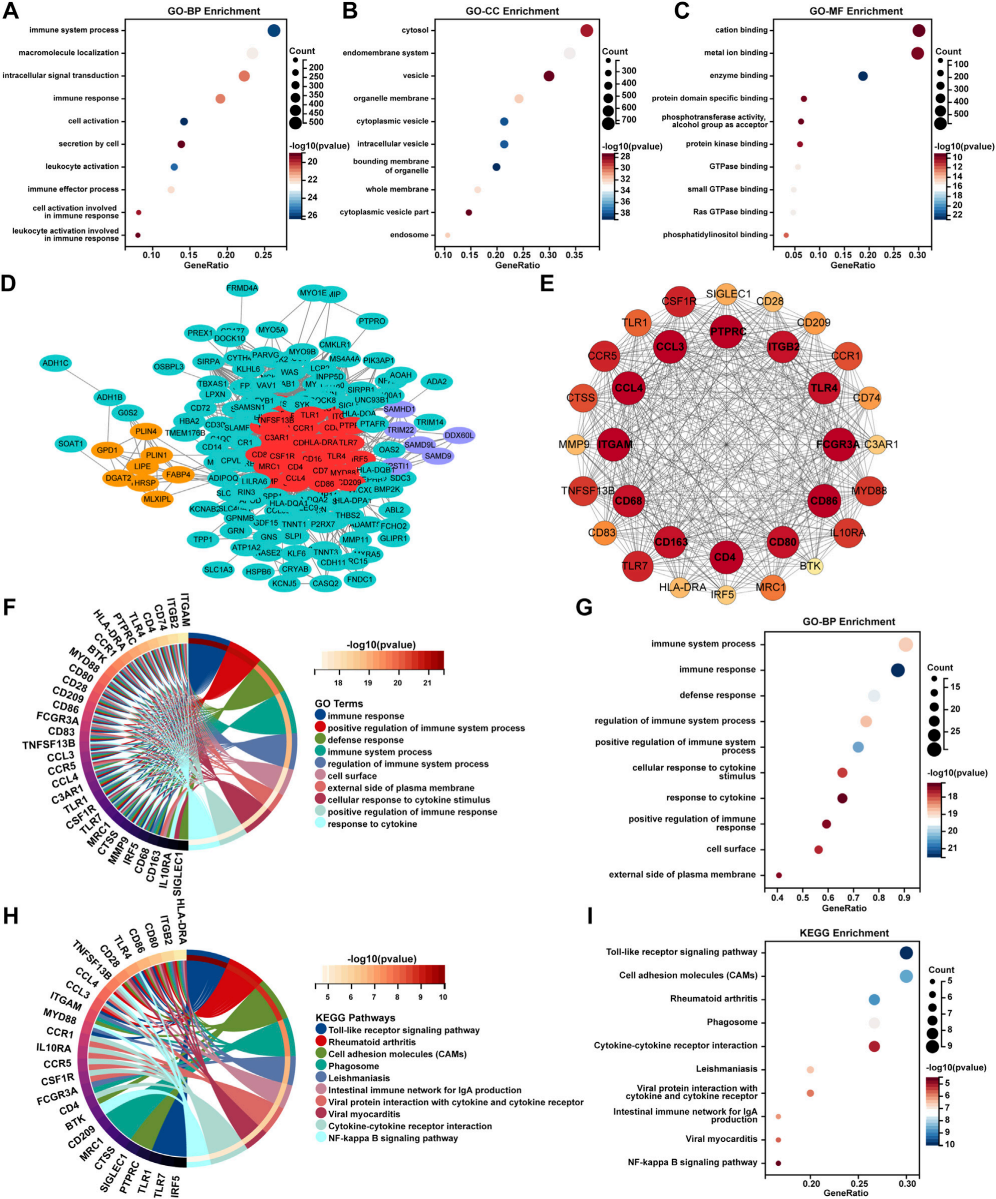
Enrichment analysis and PPI network analysis of AL samples. The bubble plots represent the results of GO enrichment analyses including **(A)** BP, **(B)** CC, and **(C)** MF for DEGs of the AL samples with an adjusted *p-*value <0.05 and FDR < 0.05. **(D)** PPI analysis of the top 250 DEGs of AL samples was performed using the STRING database, and cluster analysis was performed via the MCODE function in Cytoscape software. **(E)** The PPI network of module genes with the highest score contained 32 genes based on MCODE analysis. **(F)** Circos plot and the **(G)** bubble plot displaying the GO-BP enrichment analysis of genes included in the cluster with the highest score. **(H)** Circos plot and the **(I)** bubble plot displaying the KEGG enrichment analysis of genes included in the cluster with the highest score.

GO-BP enrichment analysis showed enrichment of these DEGs in immune-related signaling pathways such as “immune system process,” “immune response,” “leukocyte activation,” “immune effector process,” “cell activation involved in immune response,” and “leukocyte activation involved in immune response” (Figure 2A). In terms of GO-CC analysis, these DEGs were mainly enriched in “bounding membrane of organelle,” “cytoplasmic vesicle,” and “intracellular vesicle” processes (Figure 2B). GO-MF enrichment analysis suggested that these DEGs were mainly enriched in “enzyme binding,” “Ras GTPase binding,” and “small GTPase binding” processes (Figure 2C).

To further uncover the potential enrichment pathways of DEGs, PPI network analysis was performed. Cluster analysis was further carried out via the MCODE function in Cytoscape software based on the PPI network (Figure 2D; Table 2). The top gene cluster included many immune-related genes, such as *CD68*, *CD163*, *CD4*, *CD80*, and *CD86*, other immune cell markers, and inflammation-related genes such as *CCL3*, *CCL4*, *CCR1*, and *CCR5* (Figure 2E). GO-BP enrichment analysis showed that the genes included in the cluster with the highest score were mainly enriched in immune-related processes such as “immune response,” “positive regulation of immune system process,” “defense response,” “immune system process,” “regulation of immune system process,” and “positive regulation of immune response” and inflammation-related signaling pathways such as “cellular response to cytokine stimulus” and “response to cytokine” (Figures 2F, G). KEGG enrichment analysis showed that these genes were mainly enriched in “Toll-like receptor signaling pathway,” “rheumatoid arthritis,” “cell adhesion molecules,” “cytokine–cytokine receptor interaction,” and “NF-κB signaling pathway” (Figures 2H, I).

**TABLE 2:** Cluster analysis via the MCODE function in Cytoscape software.

Cluster	Score	Node	Edge	Node ID
1	27.613	32	428	*CCL3*, *PTPRC*, *CTSS*, *MRC1*, *CD163*, *TLR4*, *CD86*, *IRF5*, *TLR7*, *CSF1R*, *SIGLEC1*, *TNFSF13B*, *CD83*, *CD68*, *HLA-DRA*, *TLR1*, *CD4*, *CD74*, *CD209*, *CD28*, *CCR5*, *MMP9*, *ITGB2*, *CD80*, *ITGAM*, *MYD88*, *IL10RA*, *BTK*, *C3AR1*, *CCR1*, *CCL4*, and *FCGR3A*
2	7.143	8	25	*MLXIPL*, *PLIN1*, *GPD1*, *THRSP*, *FABP4*, *LIPE*, *DGAT2*, and *PLIN4*
3	4.8	6	12	*EPSTI1*, *SAMHD1*, *TRIM22*, *SAMD9L*, *DDX60L*, and *SAMD9*
4	4.5	5	9	*COL11A1*, *MMP11*, *THBS2*, *COL10A1*, and *LRRC15*
5	4.333	7	13	*HLA-DMA*, *DOCK2*, *PLEK*, *HLA-DPA1*, *HLA-DQB1*, *HLA-DQA2*, and *HLA-DQA1*
6	4	4	6	*TNNT3*, *SLN*, *CASQ2*, and *TNNT1*
7	3.6	6	9	*HCK*, *CD14*, *LYN*, *SYK*, *CD53*, and *LILRB2*
8	3.5	5	7	*WAS*, *DOCK8*, *CD300A*, *LCP2*, and *SASH3*
9	3	3	3	*INPP5D*, *FYB1*, and *VAV1*
10	3	3	3	*CYTH4*, *MYO1F*, and *NCKAP1L*

### Immune cell infiltration and correlation analysis of AL samples

**FIGURE 3:**
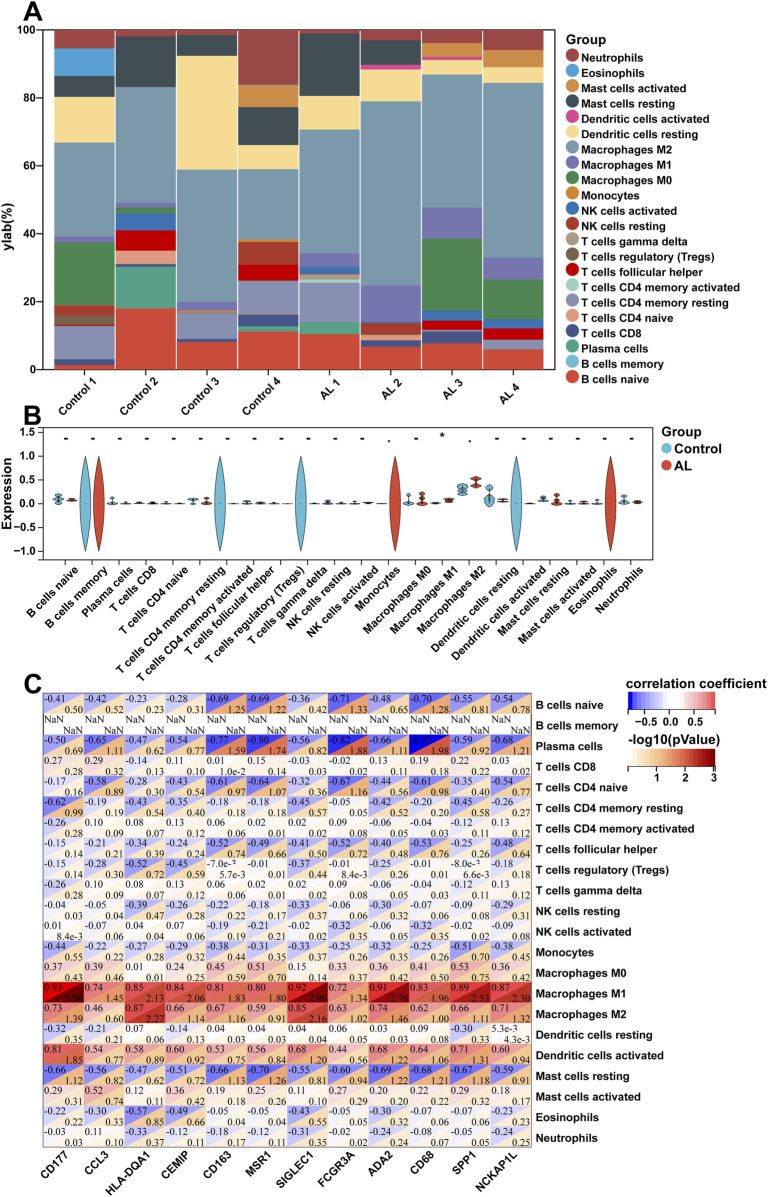
Immune cell infiltration and correlation analysis of AL samples. **(A)** Stacked histogram displaying the proportion of 22 types of immune cells between the AL and control groups. **(B)** Violin plot showing the comparison of 22 types of immune cells between the AL and control groups. **(C)** Heatmap displaying the correlation between immune-related genes in the top 20 upregulated genes and 22 immune cell type compositions. The horizontal axis demonstrates DEGs, and the vertical axis demonstrates immune cell subtypes. Data represent mean ± SD. *p < 0.05.

The enrichment analyses suggested that DEGs in AL samples were closely related to immune-related signaling pathways. Therefore, the CIBERSORT algorithm was employed to obtain the immune cell characteristics to explore the immune regulation and the correlation between DEGs and infiltrating immune cells in AL. Immunoinfiltration analysis displayed the proportion of 22 types of immune cells in each sample (Figure 3A). Compared with the control group, the proportion of M1 macrophages increased in the AL samples, whereas M2 macrophages tended to increase, but there was no statistical difference (Figure 3B). Immune-related genes in the top 20 upregulated genes were screened, and correlation analysis was performed with 22 types of immune-infiltrating cells. The correlation analysis showed that M1 macrophages were significantly positively correlated with *CD177*, *SIGLEC1*, *HLA-DQA1*, *ADA2*, *SPP1*, *CD163*, and *CD68*. M2 macrophages were significantly positively correlated with *CD177*, *HLA-DQA1*, *SIGLEC1*, *ADA2*, and *NCKAP1L*. Plasma cells were significantly negatively correlated with *CD163*, *MSR1*, *FCGR3A*, and *CD68* (Figure 3C).

### Evaluation of synovitis and validation of the expression of macrophage-associated key genes

**FIGURE 4:**
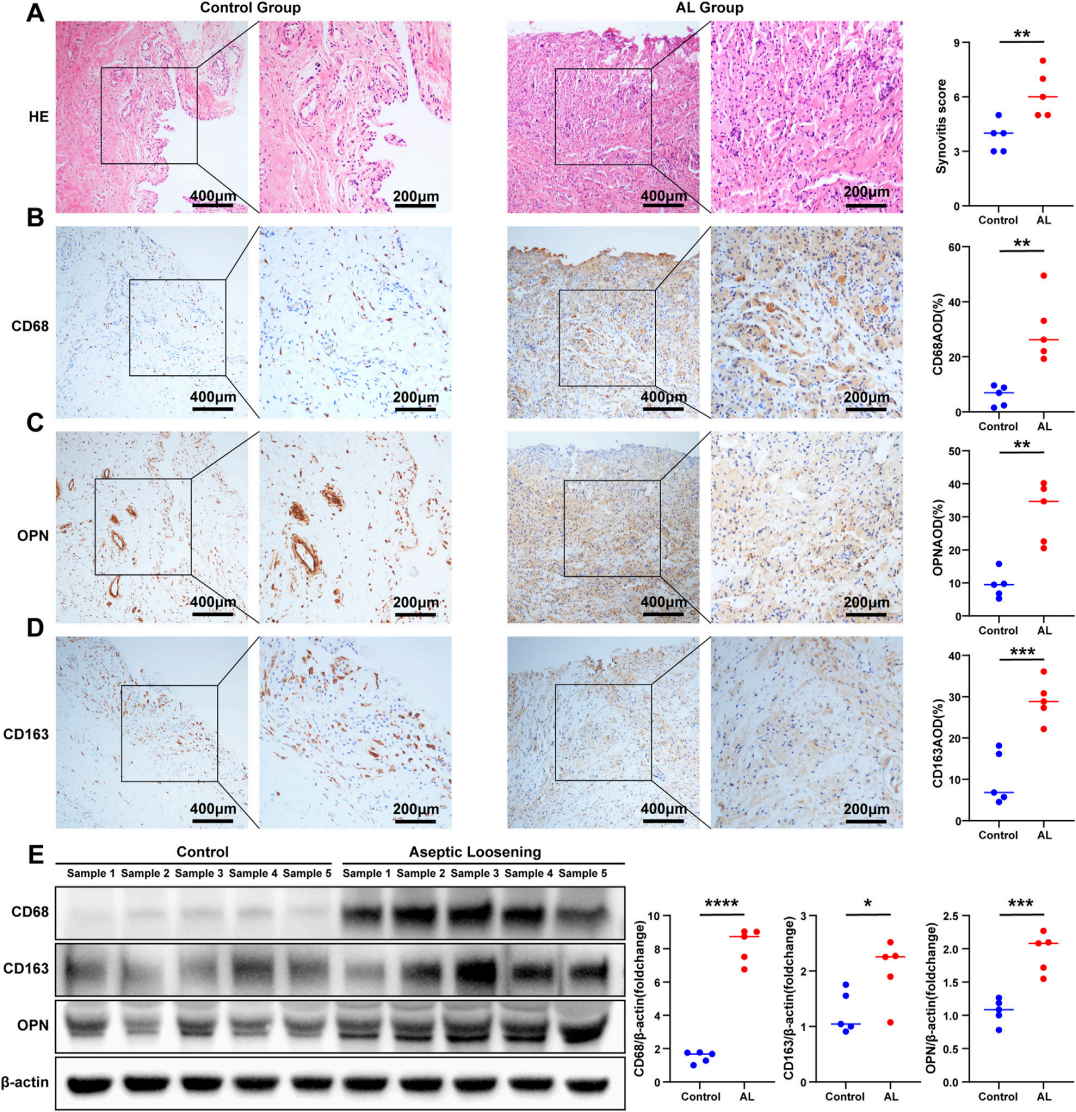
Evaluation of synovitis and validation of the expression of macrophage-associated key genes. **(A)** Representative images of HE staining of the samples in the AL group or controls (*t*-test, n = 5); scale bars, 200 μm and 400 μm. The synovitis score of the synovium was evaluated according to HE staining. Representative images of IHC staining of **(B)** *CD68*, **(C)** *OPN*, and **(D)** *CD163* in the AL and control groups (*t*-test, n = 5); scale bars, 200 μm and 400 μm. The expression of *CD68*, *OPN*, and *CD163* was quantified using ImageJ. **(E)** Western blot analysis of *CD68*, *CD163*, and *OPN* in the AL samples or controls (*t*-test, n = 5). The protein gray values were measured using AlphaEaseFC, and the expression was calculated relative to the β-actin level (*t*-test, n = 5). The data represent mean ± SD. *p < 0.05, **p < 0.01, ***p < 0.001, and ****p < 0.0001.

As bioinformatics analysis and immunoinfiltration analysis suggested significant abnormal immune and inflammatory responses in the AL group, HE staining was used to evaluate the inflammatory infiltration of synovial tissue by the synovitis scores of the two groups. The results showed that the synovitis score of the AL group was significantly higher than that of the control group (average 6.2 vs. 3.8; AL group vs. control group) (Figure 4A). In order to further explore the role of M1 macrophages in the AL group interface membrane, we screened the genes closely related to M1 macrophages in the above correlation analysis, which revealed three key genes *CD68*, *CD163*, and *SPP1* [encoding protein osteopontin (OPN)]. IHC results showed that the expression of *CD68*, *CD163*, and *OPN* in the AL group was significantly higher than that in the control group (AOD; *CD68*, average 30.03% vs. 5.85%; *CD163*, average 29.05% vs. 10.27%; *OPN*, average 31.28% vs. 9.41%; AL group vs. control group) (Figures 4B–D). Western blot analysis revealed that the expression of *CD68*, *CD163*, and *OPN* significantly increased in the AL samples compared with that in the controls (Figure 4E).

### Prediction of candidate small-molecule compounds

**FIGURE 5:**
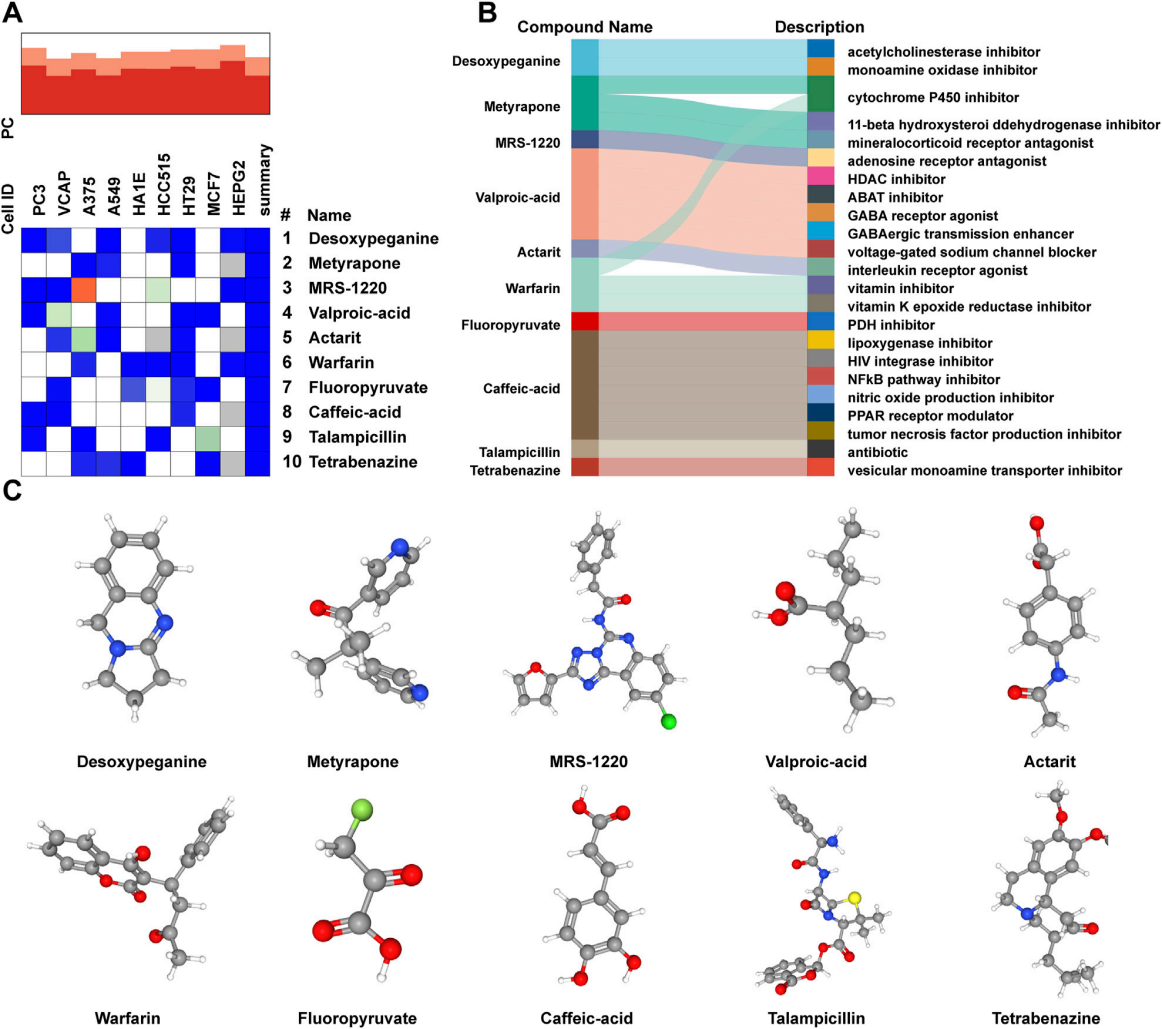
Prediction of candidate small-molecule compounds. **(A)** Heatmap presenting the top 10 compounds with the most significantly negative enrichment scores in cell lines based on CMap analysis. **(B)** Sankey diagram showing the description of the top 10 compounds. **(C)** Three-dimensional chemical structure visualization of the top 10 compounds.

To predict the potential small-molecule compounds that might play a role in therapeutic intervention of AL patients, the top 150 DEGs (all upregulated genes) were analyzed via the CMap database. The top 10 compounds with the highest negative scores were predicted to be potential therapeutic drugs for the treatment of AL: desoxypeganine, metyrapone, MRS-1220, valproic acid, actarit, warfarin, fluoropyruvate, caffeic acid, talampicillin, and tetrabenazine (Figure 5A). The Sankey diagram showed the targeted pathways of these 10 compounds (Figure 5B), and the chemical structure is visualized in Figure 5C. These compounds represent computational predictions, and their biological relevance to AL needs further *in vitro*/*in vivo* validation.

## Discussion

AL is one of the main reasons for the failure of THA and the need for revision surgery. Given the high incidence of complications, complex technical requirements, and substantial economic burden associated with revision procedures (Delanois et al., 2017; Tarazi et al., 2021), extensive research focusing on the pathogenesis of AL is urgently needed to seek more sensitive and effective diagnostic and therapeutic approaches. Our study employed various bioinformatics methodologies to delineate the transcriptomic characteristics of AL, reveal the features of immune cell infiltration, and screen potential diagnostic biomarkers, providing new insights for the diagnosis and treatment of AL patients.

The initial enrichment analysis in our study suggested that the pathogenesis of AL may focus on immune system regulation and immune cell activation. Wear particles in AL trigger dual immune responses: 1. innate immunity via macrophage phagocytosis through PRRs, releasing inflammatory cytokines and osteoclastogenic factors while causing particle cytotoxicity; 2. adaptive immunity involving lymphocyte infiltration and metal ion-induced hypersensitivity reactions (Athanasou, 2016). Histopathological analysis by Vasconcelos et al. (2016) has shown that compared to those in the osteoarthritis synovial tissue, AL patients exhibit distinct tissue architecture and immune cell profiles in the aseptic interface membrane, with significant differences in the distribution of macrophages, T cells, and B cells, and the inflammation is largely confined to the vicinity of the osteoarthritis synovium, whereas macrophages infiltrate the entire AL tissues.

Our results showed a significantly higher proportion of infiltrated M1 macrophages observed in AL patients than in control synovial tissue. Macrophages are a key population of tissue-resident mononuclear phagocytes that play essential roles in bacterial recognition and clearance, as well as in innate and adaptive immune processes (Hirayama et al., 2017). Macrophages are the predominant immune cells in the AL interface membrane and exhibit polarized phenotypes: pro-inflammatory M1 (sustaining inflammation and osteoclast activation) versus anti-inflammatory M2 (Muñoz et al., 2020). These cells phagocytose wear particles, forming multinucleated giant cells and triggering bone resorption (Hodges et al., 2021; Nich et al., 2013).

Additionally, the study by Panez-Toro et al. (2023) discussed the role of non-myeloid monocytes in the periprosthetic tissue exposed to wear particles, including the coexistence of T cells (CD3^+^, CD4^+^, and CD8^+^) and B cells (CD20^+^) with CD68^+^/TRAP^−^ multinucleated giant cells associated with polyethylene and metal particle infiltration of the repair tissue membrane. Our study also found that T-cell-related markers, including *CD4*, *CD28*, *CD80*, *CD86*, and *CD53*, and B-cell-related markers, including *CD72* and *CD83*, were significantly upregulated in AL. T cells play dual roles: regulatory T cells inhibit osteoclastogenesis via IL-4 (Kim et al., 2007), whereas Th1 cells promote macrophage activation (Wu et al., 2022). Metal particles may induce T-cell hypersensitivity reactions (Niki et al., 2005). B-cell infiltration suggests adaptive immunity activation, though typically more prominent in infections.

Macrophage polarization is regulated by multiple pathways, primarily through Toll-like receptors (TLRs; e.g., TLR2/4), which activate NF-κB via MyD88/IRAK/TRAF6 signaling (Jang et al., 2017). TLR2 is notably expressed in periprosthetic tissues and mediates local inflammation (Naganuma et al., 2016). NF-κB activation (by LPS/RANKL) promotes M1 polarization and osteoclastogenesis (Maehara and Fujimori, 2020; Liu et al., 2019); however, MAPK, JAK/STAT, and Ca^2+^ pathways also contribute to the process (Cong et al., 2023). The enrichment analysis in our study also showed that DEGs were mainly enriched in the “Toll-like receptor signaling pathway” and “NF-κB signaling pathway.” Consequently, the abnormal regulation of the immune response process and immune cells (especially macrophage polarization) may be the primary pathogenic mechanism of AL.

To further explore the role of macrophages in the interface membrane of the AL group, three key genes closely related to macrophages, namely, *CD68*, *CD163*, and *SPP1* [encoding osteopontin (OPN)], were screened. IHC and Western blotting results showed higher levels of *CD68*, *CD163*, and *SPP1* in AL, providing potential novel serum biomarker candidates for the diagnosis of AL. Resident macrophages in the synovium are identified as CD68^+^ and CD163^+^ cells, which remain relatively quiescent but become activated during disease flares. CD68 is a highly glycosylated glycoprotein that is highly expressed in macrophages and other mononuclear phagocytes and used as a valuable cytochemical marker for immunostaining of monocytes/macrophages in inflammatory tissues, tumor tissues, and other immunohistopathological applications (Chistiakov et al., 2017). In the treatment of rheumatoid arthritis, changes in the number of CD68^+^ macrophages are associated with clinical outcomes assessed by DAS28 and are considered a reliable biomarker for assessing the efficacy of rheumatoid arthritis treatments (Haringman et al., 2005). CD163 is a hemoglobin scavenger receptor that is highly expressed in tissue-resident macrophages, aids in anti-inflammatory local responses, reduces hemoglobin levels, and promotes anti-inflammatory heme metabolism products (Kristiansen et al., 2001).

Osteopontin (OPN), encoded by *SPP1*, is a multifunctional glycoprotein secreted by immune cells (macrophages and T lymphocytes) and present in inflammatory and mineralized tissues (Si et al., 2020). It mediates osteoclast adhesion via αvβ3 integrin/CD44 interactions and recruits immune cells to inflammation sites (Gravallese, 2003). Studies have shown that OPN regulates osteoclastogenesis through NF-κB and cytokine modulation (Icer and Gezmen-Karadag, 2018), with macrophage-expressed OPN influencing inflammation through cytokine production and phagocytosis (Rittling, 2011). Shimizu et al. (2010) showed that OPN expression was enhanced in human periprosthetic osteolysis tissues compared to osteoarthritis synovial tissues. In the particle-induced model of calvarial osteolysis, bone resorption was significantly suppressed by OPN deficiency through the inhibition of osteoclastogenesis. Tang et al. (2023) showed that OPN regulated pro-inflammatory cytokines and promoted macrophage polarization toward the M1 phenotype in rosacea-like skin inflammation. Similarly, our study found *SPP1* to be highly expressed in AL, indicating that activated macrophages may regulate osteoclast function through the secretion of OPN and participate in the pathological process of AL.

Our study also has the following limitations: first, the sample size of this study is small, and we are currently expanding the cohort for future validation. Second, our study used PFF patients as controls, which may not fully represent a healthy synovial state. Fracture-related acute inflammation or trauma-induced biological processes could introduce confounding effects. Future studies should validate our findings using additional healthy control groups. Third, although our connectivity graph analysis identified potential compounds, their efficacy and safety in the treatment of AL are still needed to be tested in future studies. Therefore, future studies should prospectively compare AL that occurs after THA arising from diverse etiologies. Finally, although our samples were derived from the liner–ball–neck interface, future studies should analyze direct bone-implant membranes.

## Conclusion

This study based on AL interface membrane and PFF synovial samples demonstrated the gene characteristics of AL through transcriptomic and integrated bioinformatics analyses and preliminarily identified *CD68*, *CD163*, and *SPP1* as potential biomarkers for AL, providing new insights for the diagnosis and treatment of AL.

## Data Availability

The datasets presented in this study can be found in online repositories. The names of the repository/repositories and accession number(s) can be found in the article/Supplementary Material.
